# Supplementary material for: Compound Eye Structure and Phototactic Dimorphism in the Yunnan Pine Shoot Beetle, Tomicus yunnanensis (Coleoptera: Scolytinae)
Source: Biology (Basel). 2025 Aug 11;14(8):1032. doi: 10.3390/biology14081032 (PMC12383413; doi:10.3390/biology14081032)

Supplementary Materials

Figure S1. Corneal ultrastructure in *T. yunnanensis* compound eyes. (A) Longitudinal section through the central region showing toluidine blue staining differences between the outer lens unit and inner lens unit. (B) Transverse section of the proximal cornea (ILU), revealing its structural continuity and connections. (C) Transverse section at the ILU-cone cell interface, surrounded by primary and secondary pigment cells. (D) Enlarged view of the ILU center: microfibrils splay laterally, converge towards the ommatidial axis core, and coil around it to form a spiral structure. BM, basal matrix; CC, cone cell; COR, cornea; ILU, inner lens unit; OLU, outer lens unit; NCSPC, nucleus of secondary pigment cell; PG, pigment granules; PPC, primary pigment cells; RBD, rhabdom; SPC, secondary pigment cell. Scale bars: (A) 50 μm; (B) 10 μm; (C, D) 1 μm.

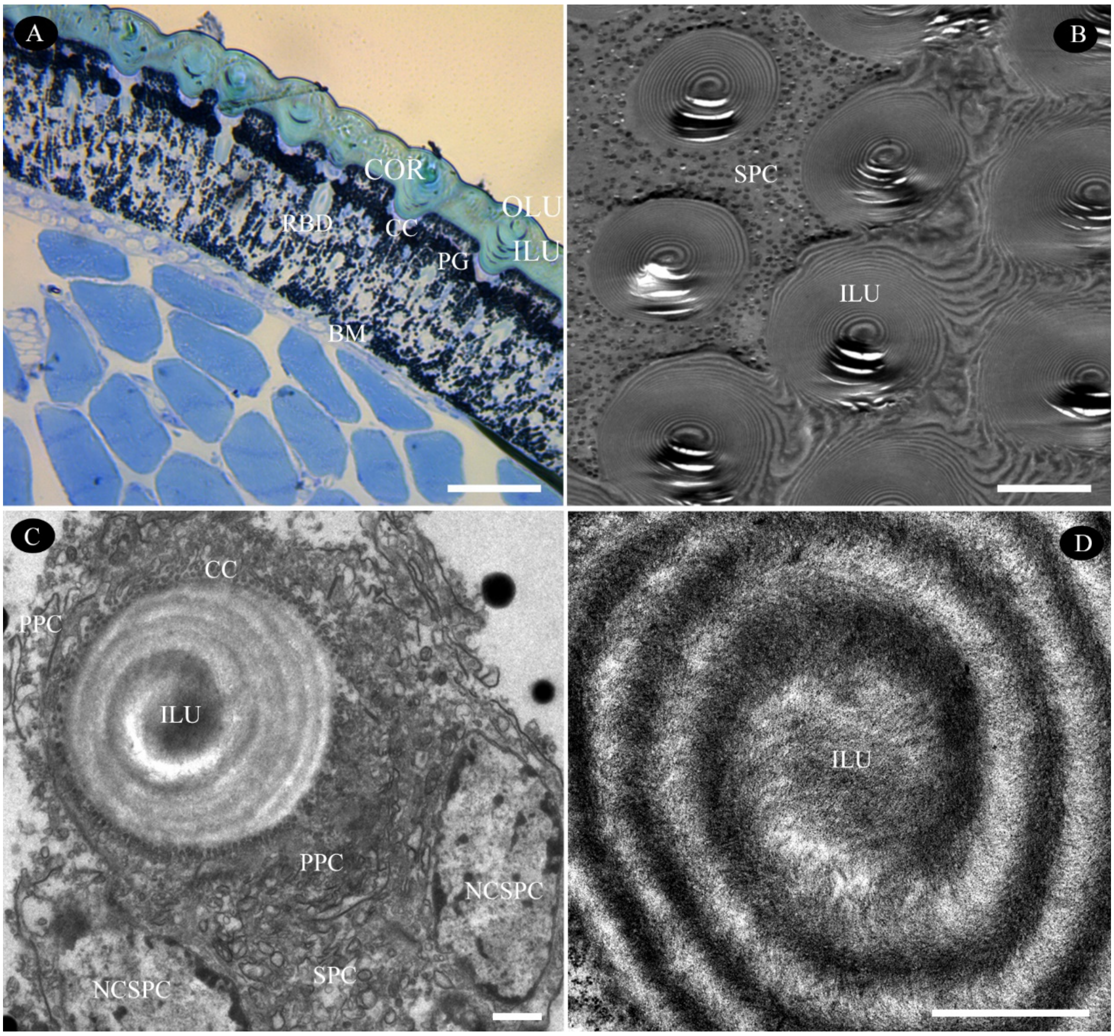

Figure S2. Cone ultrastructure in *T. yunnanensis* compound eyes. (A) Longitudinal section of the proximal cone, showing its direct connection (arrowhead) to the rhabdomere. (B) Dense aggregations of virus-like particles (VLPs) adjacent to cone cell nuclei within ommatidia. (C) Enlarged view of VLPs. CR, central rhabdomere; NC, nucleus; PR, peripheral rhabdomere; VLPs, virus-like particles. Scale bars: (A, B) = 1 μm; (C)=500 nm.

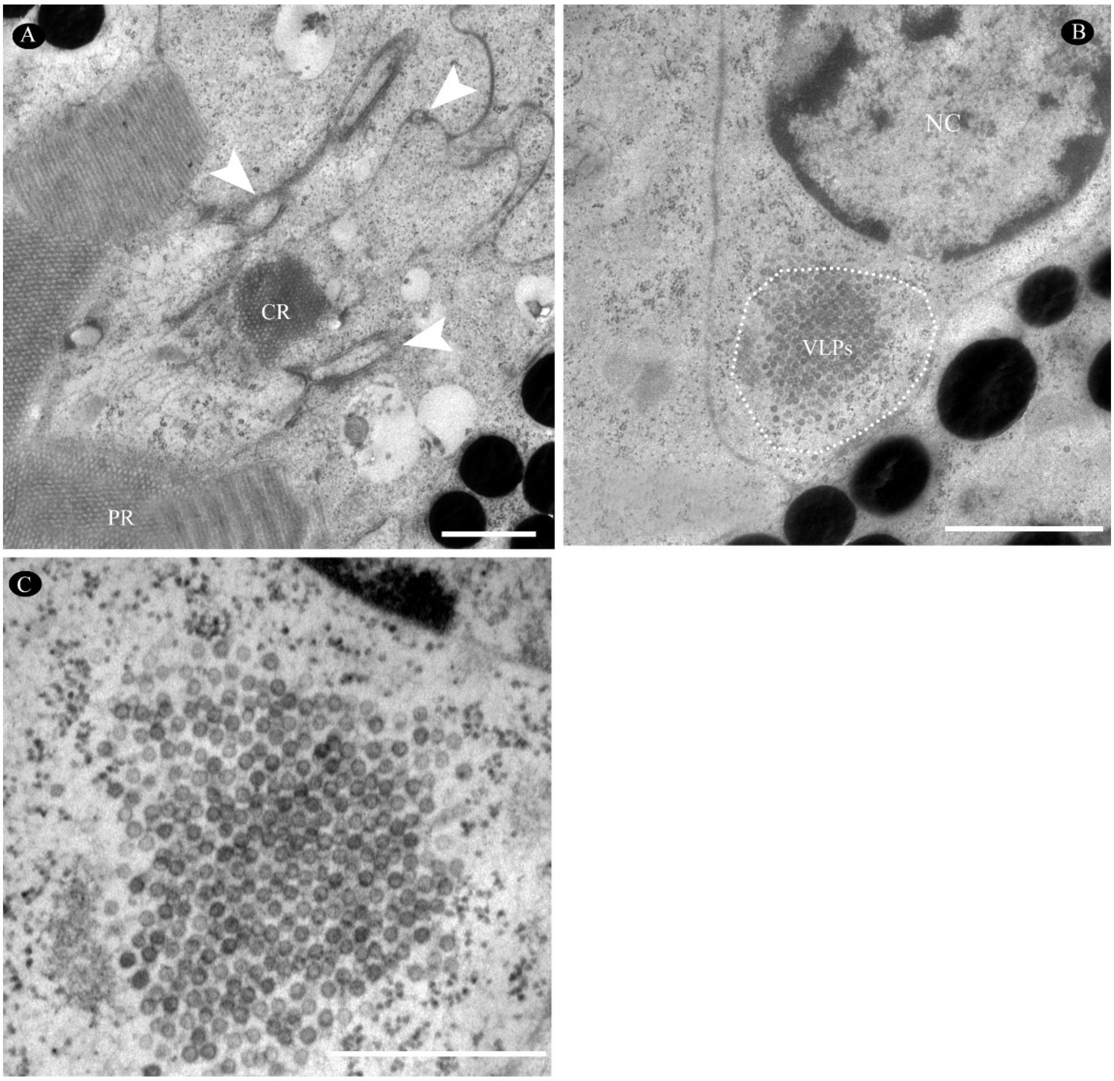

Figure S3. Transverse sections of ommatidial facets in *T. yunnanensis*. (A) Dorsoventral overview. Boxed regions (capital letters) indicate areas enlarged in subsequent panels. Anatomical orientation: a, anterior; p, posterior; v, ventral; d, dorsal. (B) Hexagonal facet. (C) Rectangular facet. (D) Cytoplasmic organelles within retinular cells, showing endoplasmic reticulum (EPR), mitochondria (MT), multivesicular bodies (MVB), pigment granules (PG), and retinular cell nucleus (RNC). Scale bars: (A) = 20 μm; (B-D) =1 μm.

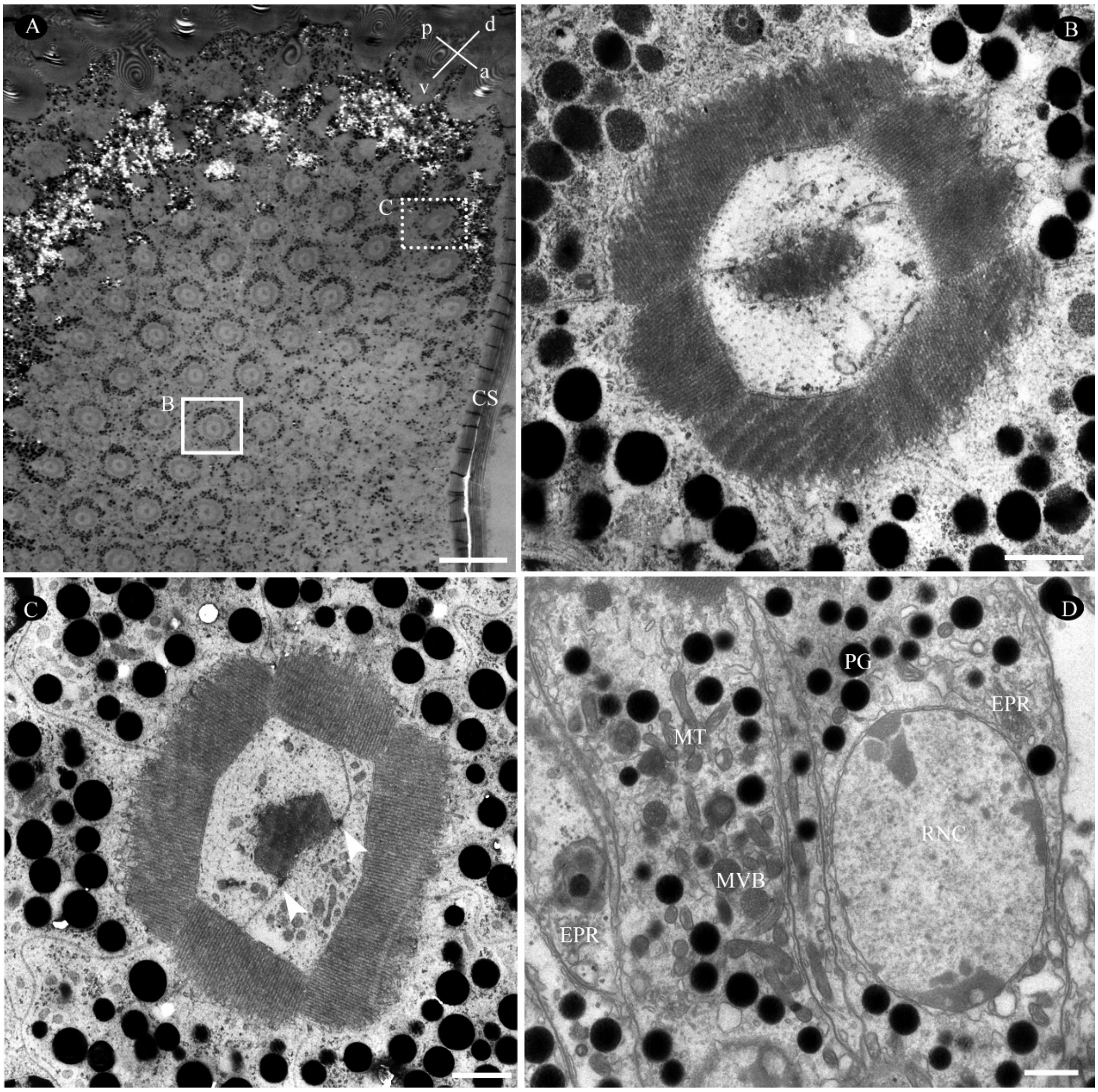

Figure S4. Transverse sections of the basal matrix in *T. yunnanensis*. (A) Overview showing multiporous structures and containing large nuclei, mitochondria, and pigment granules. (B) Tracheoles above the basal matrix extend distally through interommatidial spaces. (C) Enlarged view detailing axon bundles (each containing eight axons, AX); arrowhead indicates an axon penetrating the BM. AX, axons; BM, basal matrix; LAM, lamina; MT, mitochondria; NC, nucleus; PSL, photosensitive layer; TR, tracheole. Scale bars: (A) =10 μm; (B, C) =1 μm.

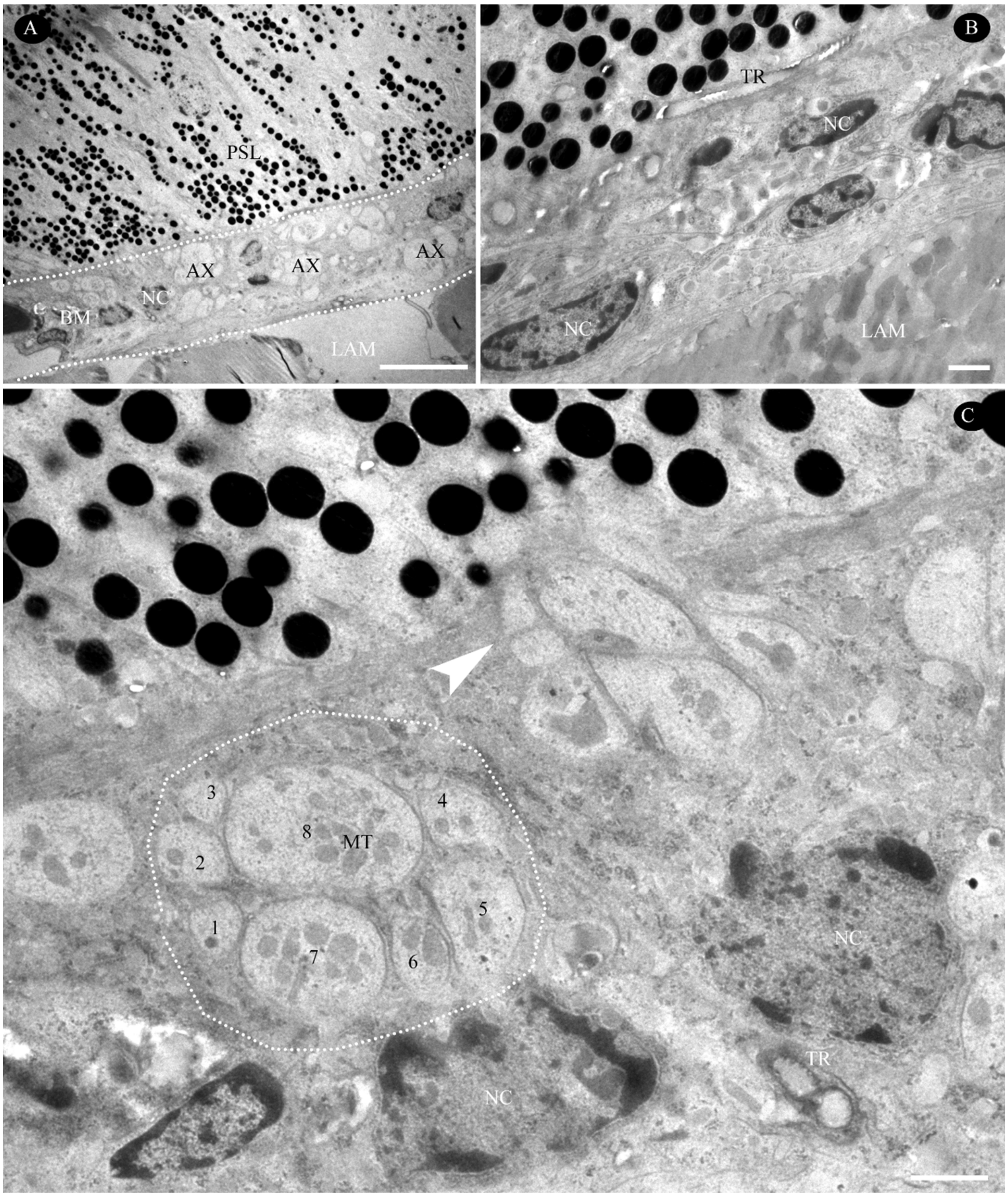

Supplement: Supplementary file 1 [file biology-14-01032-s001.zip › biology-3787319-supplementary.pdf]
